# Public health round-up

**DOI:** 10.2471/BLT.17.010917

**Published:** 2017-09-01

**Breastfeeding brings health and development gains**

A child breastfeeding in Bijulidanda of the Gorkha district in Nepal – one of only 23 countries worldwide where more than 60% of newborn babies are exclusively breastfed during the first six months.

**Figure:**
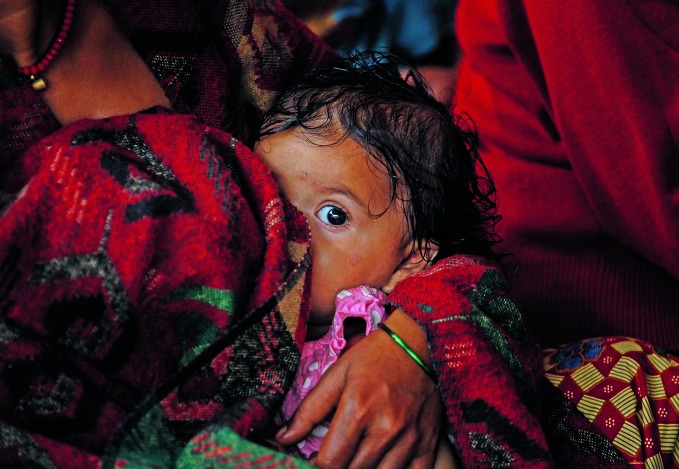
©UNICEF/UNI192899/Shresth

## Call for more investment in breastfeeding

Many societies are failing to adequately support women to breastfeed their babies, and as a result, most of the world’s children do not reap the full benefits of breastfeeding, according to two new reports.

The first report, entitled *Nurturing the health and wealth of nations: the investment case for breastfeeding*, was developed by the United Nations Children’s Fund (UNICEF) and the World Health Organization (WHO) in collaboration with the Global Breastfeeding Collective, a new initiative launched last month to increase breastfeeding globally.

Governments need to invest more in the promotion of breastfeeding, including paid family leave and workplace breastfeeding policies, and full implementation of the *International code of marketing of breast-milk substitutes*, according to the report.

The authors estimated that an investment of US$ 4.70 per newborn to promote breastfeeding could save the lives of 520 000 children and generate economic returns of US$ 300 billion across low- and middle-income countries by 2025.

Investment in breastfeeding globally is low. Each year, governments in low- and middle-income countries spend some US$ 250 million on breastfeeding promotion while donors provide only an additional US$ 85 million.

In the second report, *Global breastfeeding, 2017: tracking progress for breastfeeding policies and programmes*, WHO and UNICEF found that no countries are adequately protecting, promoting or supporting breastfeeding through funding or policies.

An estimated 40% of all children younger than six months are breastfed exclusively (given nothing but breastmilk), and exclusive breastfeeding is above 60% in only 23 of 194 countries.

Breastfeeding helps to prevent diarrhoea and pneumonia, two major causes of death in infants, and reduces the risk of ovarian and breast cancer in mothers.

The campaign was launched during World Breastfeeding Week from 1–7 August.

http://www.who.int/mediacentre/news/releases/2017/lack-investment-breastfeeding

## Making hepatitis C treatment affordable

WHO has prequalified the first generic version of sofosbuvir, a first-line medicine for the treatment of hepatitis C, putting it within reach of the millions of people in need in low- and middle-income countries.

The “prequalified” stamp of approval means that this generic version of sofosbuvir is safe, effective and of high quality, and is thus recommended for bulk purchase by United Nations agencies and funding agencies such as UNITAID.

Hepatitis C is a bloodborne virus that can cause a chronic illness leading in some cases to liver cirrhosis and cancer.

Globally, an estimated 71 million people have chronic hepatitis C infection and some 399 000 people die each year from hepatitis C, mostly from the complications of liver cirrhosis and hepatocellular carcinoma.

In response to the global epidemic, several countries are already procuring generic versions of sofosbuvir, including Cambodia, Egypt, Ethiopia, Indonesia, Kenya, Mongolia, Myanmar, Nepal, Pakistan, Rwanda, Uganda, Viet Nam and Zambia.

Sofosbuvir, in the form of a 400 mg tablet, is manufactured by Indian pharmaceutical company Mylan Laboratories Ltd. Its prequalification is expected to lead to an expansion of treatment for many more people who need it, but cannot afford it.

A three-month treatment course with Mylan’s sofosbuvir costs as little as US$ 260, a fraction of the medicine’s market entry price of US$ 28 000 a month in 2013 and of the price set for a month’s course in most high-income countries.

Antiviral medicines can cure more than 95% of people with hepatitis C infection, thus reducing the risk of death from liver cancer and cirrhosis.

## Delivering medicines and vaccines to Syrians

WHO delivered life-saving medicines last month to the Syrian city of Al-Qamishli for thousands of displaced people from the governorates of Al-Raqqa, rural Deir Ez-zor and Al-Hassakeh in the north-east of the Syrian Arab Republic.

It was the first time since 2014 that WHO staff could reach the city by road, after fighting in the area subsided in recent weeks. WHO delivered almost 30 tonnes of medicines and medical supplies, enough for 150 000 treatments, for health facilities and mobile clinics in the northern and eastern regions of the country on 1 August.

In a separate consignment at the end of July, WHO delivered more than half a million doses of polio vaccine for an emergency campaign that succeeded in reaching some 260 000 children in Deir Ez Zor. Another campaign targeting 120 000 children aged under five years was planned in rural Al-Raqqa last month. 

The two emergency campaigns aimed to stop vaccine-derived poliovirus type-2 circulating in the area and prevent further spread to other conflict-affected areas, where children have not had regular access to polio vaccination.

Thirty cases of circulating vaccine-derived poliovirus type-2 have been confirmed in the country in recent years, 29 of them in Deir Ez-Zor and one in rural Al-Raqqa. The last reported case of wild poliovirus in the country was in January 2014.

In June, WHO delivered more than 12 000 treatments for people with chronic, infectious and diarrhoeal diseases to the two main camps for internally displaced persons in rural Al-Raqqa, one of the governorates in the north east of the country. Al Karameh camp hosts more than 35 000 children, women and men and Ain-Issa camp hosts more than 3500 people.

http://www.emro.who.int/syr/syria-news/who-delivers-medicines-and-medical-supplies-to-al-qamishli.html

## HIV self-test prequalifed

WHO has prequalified the first self-test for HIV infection, paving the way for countries to purchase these tests in bulk so that more people can check their HIV status.

In 2016, an estimated 30% of all people living with HIV remained unaware of their HIV status, many of these people are from high-risk populations who are either less likely to approach a health facility or are unable to do so.

The product, OraQuick® HIV Self-Test, manufactured in Thailand for OraSure Technologies Inc., uses saliva as a specimen and provides results in about 20 minutes.

Prequalification of OraQuick ® HIV Self-Test means that it is recommended for United Nations agencies and funding agencies for bulk purchase, especially for countries with weak laboratory infrastructure and limited capacity to do HIV testing.

In 2016, WHO recommended HIV self-testing as a complementary approach to reach people who are afraid to come forward for a formal diagnosis and treatment for fear of stigma and discrimination.

“Over the past year, the number of countries incorporating HIV self-testing into their policies has increased from 16 to 40. This is impressive progress,” said Dr Gottfried Hirnschall, Director of the HIV Department at WHO in Geneva.

Most international funding agencies have pledged their support for the procurement and deployment of HIV self-tests, including a specific agreement on affordable pricing for 50 lower middle-income countries between the manufacturer and the Bill and Melinda Gates Foundation.

WHO prequalification assures the quality, safety and efficacy of priority medicines, vaccines and in vitro diagnostics bought by international procurers for low-income countries.

http://www.who.int/medicines/news/2017/1st_generic-hepC_1stHIVself-test-prequalified

**Cover photo**

Two refugees who fled gang violence in El Salvador with their families and live in Chiapas, Mexico. Their family has received refugee status in Mexico, but their parents have had problems finding work and being accepted by the local community.

**Figure:**
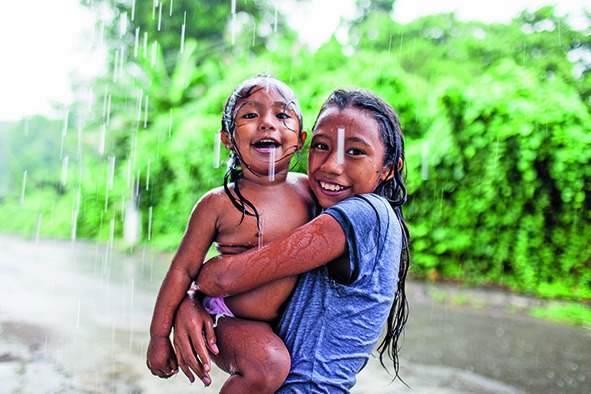
© UNHCR/Markel Redondo

## New WHO guidelines on HIV

WHO has released new guidelines that programme managers, clinicians and others can use to address the needs of people with advanced HIV disease and to decide how soon anyone living with HIV infection should start treatment.

The new publication entitled, *Guidelines for managing advanced HIV disease and rapid initiation of antiretroviral therapy*, comprises two sets of recommendations.

The first set outlines health-care interventions for people with advanced HIV disease to reduce the risk of HIV-associated disease and death. These include screening, prophylaxis, rapid initiation of antiretroviral therapy and intensified adherence interventions for people with advanced disease.

One of these interventions – the rapid initiation of antiretroviral therapy – is covered in detail by the second set of recommendations. Rapid antiretroviral therapy initiation should be offered as soon a person’s HIV diagnosis is confirmed. This should be as early as the same day – depending on the person’s readiness to start immediately – or within seven days of diagnosis, according to the new guidelines.

Advanced HIV disease is defined as a CD4 cell count <200 cells/mm3 or a WHO clinical stage 3 or 4 event. In 2015, more than a third (37%) of HIV-positive individuals who started antiretroviral therapy had advanced HIV disease.

http://www.who.int/hiv/pub/guidelines/advanced-HIV-disease

**Looking ahead**

13–16 September – Global Evidence Summit, Cape Town, South Africa

18–20 October – WHO Global Conference on Noncommunicable Diseases. Montevideo, Uruguay

1–3 November – World Hepatitis Summit 2017. São Paulo, Brazil

16–17 November – Global Ministerial Conference on Tuberculosis. Moscow, Russian Federation

